# Clinical Trial of a Home Safety Toolkit for Alzheimer's Disease

**DOI:** 10.1155/2013/913606

**Published:** 2013-09-29

**Authors:** Kathy J. Horvath, Scott A. Trudeau, James L. Rudolph, Paulette A. Trudeau, Mary E. Duffy, Dan Berlowitz

**Affiliations:** ^1^VA New England Geriatric Research Education & Clinical Center, Bedford, MA 01730, USA; ^2^Edith Nourse Rogers Memorial Veterans Hospital, Bedford, MA 01730, USA; ^3^Alzheimer's Disease Center, Boston University School of Medicine, Boston, MA 02118, USA; ^4^VA Center for Health Quality Outcomes and Economics Research, Bedford, MA 01730, USA; ^5^Department of Occupational Therapy, Tufts University, Medford, MA 02155, USA; ^6^VA Boston Healthcare System, Boston, MA 02130, USA; ^7^Massachusetts General Hospital Yvonne L. Munn Center for Nursing Research, Boston, MA 02114, USA

## Abstract

This randomized clinical trial tested a new self-directed educational intervention to improve caregiver competence to create a safer home environment for persons with dementia living in the community. The sample included 108 patient/caregiver dyads: the intervention group (*n* = 60) received the Home Safety Toolkit (HST), including a new booklet based on health literacy principles, and sample safety items to enhance self-efficacy to make home safety modifications. The control group (*n* = 48) received customary care. Participants completed measures at baseline and at twelve-week follow-up. Multivariate Analysis of Covariance (MANCOVA) was used to test for significant group differences. All caregiver outcome variables improved in the intervention group more than in the control. Home safety was significant at *P* ≤ 0.001, caregiver strain at *P* ≤ 0.001, and caregiver self-efficacy at *P* = 0.002. Similarly, the care receiver outcome of risky behaviors and accidents was lower in the intervention group (*P* ≤ 0.001). The self-directed use of this Home Safety Toolkit activated the primary family caregiver to make the home safer for the person with dementia of Alzheimer's type (DAT) or related disorder. Improving the competence of informal caregivers is especially important for patients with DAT in light of all stakeholders reliance on their unpaid care.

## 1. Introduction

Dementia of the Alzheimer's type is a growing public health problem. An estimated 35.6 million people were living with dementia in 2010, and the number is expected to double every 20 years to 65.7 million in 2030 and 115.4 million in 2050 [1]. In the United States, an estimated 5.2 million people have dementia of the Alzheimer's type (DAT), a number that is projected to grow to 13.8 million by 2050 [2]. Other population studies estimate that 13% or 1 in 8 Americans aged 71 or older suffers from this disease [3], the sixth leading cause of death in the United States for which there is no treatment or cure [4]. A person with DAT will live an average of four to eight years and as long as 20 years after the onset of symptoms [5] during which 80% of their care is provided by family and friends [6]. These family caregivers absorb the largest costs of care in both dollars and emotional distress. In high-income countries, informal care accounts for 45% of the worldwide costs of dementia, $604 billion USD, and, in low-income and lower-middle-income countries, the costs of informal (unpaid) care by family caregivers account for the majority of costs [7]. Further, because of the demands of caring for a person with DAT, family caregivers have negative health consequences and increases in health care costs for themselves [8, 9]. The currently incurable nature of dementia of the Alzheimer's type, the duration of the illness, the suffering it can cause to patients and their families, and the cost of care make providing safe community care a priority. 

Health-related safety in the home and community is a public health concern for all individuals, especially older adults striving to “age in place” [10]. A review of empirical evidence regarding home safety of people across the age span and with or without disability revealed that home modifications increase functional ability outcomes although inconsistencies in study results and limitations in research designs exist [11]. The authors emphasized the importance of future studies to examine not only the physical environment but also the impact of intraindividual factors and the social environment of the home on functional ability. These authors note the relatively little home safety research that has been conducted with people who have dementia-related disorders.

Older adults are at increased risk of accidents and injuries in the home compared to the general population. Among those over the age of 65 in the United States, accidental injuries are the ninth leading cause of death, in particular fatality due to falls and fire; common nonfatal injuries include falls, being struck by objects, motor-vehicle incidents, and lacerations [12]. Persons with dementia are at even greater risk than their peers because of the cognitive and functional impairments associated with the illness [13, 14]. Prior research has revealed risks for injury for persons with dementia living at home that can be categorized as either high frequency risks, high severity risks, or both: falling, cooking, wandering, driving, home fires, medication problems, and unsafe firearms and sharps [15–17]. For example, general wandering around the home may be considered a high frequency risk for injury (i.e., common but not necessarily dangerous), whereas nighttime wandering in inclement weather is a less frequent but more severe risk. Even in early stages of memory loss, if a person is unaware of memory loss, they are more likely to have difficulty with everyday decisions such as medication management [18]. In addition, patients' self-evaluations of their ability to conduct instrumental activities of daily living (IADL) such as medication management and ability to use the telephone are significantly higher than caregivers' objective evaluations [19]. 

Not surprisingly, home safety is an immediate concern for families when a member is diagnosed with DAT. In a recent descriptive study of 82 older adults with dementia living in the community, the authors identified the prevalence of home safety modifications as well as barriers to implementation [20]. Marquardt and colleagues found 44% of homes had outdoor steps with no railings; only 23% had modified bathrooms with at least three safety components: grab bars, walk-in shower, and shower seat; 12% of homes were rated as very cluttered, which did not allow for safe mobility inside the house. These authors also found caregivers attributed almost three times more home modifications to physical impairments as opposed to memory loss in their loved ones; this suggests that caregivers may lack insight into how cognitive deficits impact the way people with dementia navigate their environments [20]. Caregivers have identified several barriers to making the home safer; in particular, lack of knowledge and impractical options were identified; conversely, trained professionals can facilitate the implementation of home safety modifications by providing caregivers with practical information and advice about what to do [21, 22].

**Figure 1:**
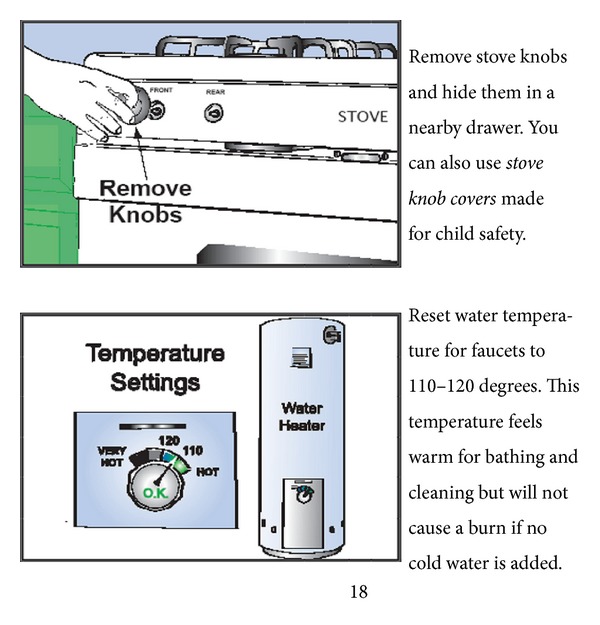
Example of recommendations in Home Safety Toolkit using health literacy principles.

This randomized controlled trial (RCT) continues a program of research guided by the Home Safety/Injury Model [15, 21, 23] which aims at giving informal caregivers the knowledge and resources to prevent risky behaviors and accidents in the homes of persons with dementia of the Alzheimer's type (DAT) or a related dementia. Our initial studies revealed that family caregivers were overwhelmed by the potential for safety hazards in the home and did not know where or how to make the most important home modifications. Even in the presence of adequate skills, however, a person's perception of low self-efficacy and self-doubts about the ability to perform a task can interfere with achievement [24]. Thus, a Home Safety Toolkit was designed to focus on improving caregiver competence by increasing practical abilities through a health literacy tested booklet that focuses on the high frequency/high severity risks for accidents and injuries in the home (Figure 1). In addition, the Home Safety Toolkit (HST) includes sample items (Table 1) which allow the caregiver to immediately and easily make home safety modifications, increasing caregiver self-efficacy through enactive attainment—theorized to be the most powerful of the influences on self-efficacy [25]. Our initial studies used an intensive professional assessment/intervention by a registered nurse (RN) and an occupational therapist. In contrast, this current HST, informed by health literacy principles, is specifically designed to be a self-directed, self-paced intervention for the caregivers which allows them to decide when and how to make modifications in the environment. 

Experience from prior studies informed the generation of hypotheses for this RCT. For instance, we previously found correlations between caregiver self-efficacy and years of formal education and perceived social support which lead to the decision to include these variables as covariates in the hypothesis testing: *Hypothesis 1. *After controlling for the effects of caregiver years of formal education, baseline caregiver self-efficacy, baseline caregiver strain, and social support, caregivers who receive the Home Safety Toolkit intervention will have higher postintervention self-efficacy, lower postintervention caregiver strain, and improved environmental home safety than caregivers in the control group who receive standard patient education: *Hypothesis 2.* After controlling for the effects of caregiver years of formal education, baseline caregiver self-efficacy, baseline caregiver strain, and social support, care recipients who receive the Home Safety Toolkit intervention will have fewer risky behaviors and accidents when compared to the care recipients in the control group who receive standard patient education.

## 2. Methods

### 2.1. Participants

Study participants were recruited from the Bedford, Massachusetts Veterans Administration Medical Center (VAMC), VA Boston Healthcare System (VABHS), and Boston University Alzheimer's Disease Center (ADC). Participants were dyads of primary caregivers and persons with a progressive dementia of the Alzheimer's type who lived in the community, were willing to have home visits for home safety education, and read and spoke English. 

Inclusion criteria for care recipients were diagnosis of dementia of the Alzheimer's type or a related disorder, score of 24 or less on the Mini-Mental State Examination [26], expected to continue living in the community for the next 6 months, and having the ability to ambulate without help from the caregiver (assistive devices to self-ambulate were acceptable). Inclusion criteria for the primary informal caregiver were living in the home with the care recipient, provid a minimum of 4 hours of caregiving per day, and have no known or apparent cognitive impairment upon screening. Exclusion criteria were a previous home safety visit and admission to a long-term care facility. Persons with DAT who were living alone were excluded because their safety issues are more complex and there is no primary informal caregiver who can make consistent observations about risky behaviors and accidents. 

### 2.2. Procedures

The study was reviewed and approved by the Institutional Review Boards (IRB) of Bedford VAMC, VA Boston Healthcare System, and Boston Medical Center and monitored semiannually by a Data Safety Monitoring Board of VA Health Services Research and Development as required for multisite studies. Potential subject dyads were enrolled from geriatric specialty clinics. Two sites, the Bedford Dementia Clinic and the Boston University Alzheimer's Disease Center (ADC), maintain a registry of families who have agreed to participate in research. At the Boston University ADC, the recruitment coordinator sent a postcard to eligible caregivers who returned the card to indicate whether they agreed to be contacted by one of the researchers [27]. In addition, because this “Opt-In” strategy can lead to a more self-selected group of participants, the recruitment coordinator also telephoned eligible participants if they neglected to return the postcard. At Bedford VAMC, unless a family “opted out” of all research, they were sent an introductory flier (approved by the IRB) with a follow-up phone call by the project director or research assistant. At VA Boston Geriatric Consultation Clinics, the research staff was available onsite to give the flier to potential patient/caregiver dyads and requested a follow-up phone call to discuss the project in more detail. Thus, subject dyads were essentially self-referred rather than referred by a clinical provider, an approach that was favored by clinical providers who did not want to use their limited clinical time to discuss research. The randomization method was stratified by site, and a permuted-block randomization was used in order to balance the number of patients assigned to each group [28]. Computer-generated random numbers were used by the statistician to allocate group assignment by the sealed envelope method [29].

Home visits were used for the initial and final data collection because in our preliminary studies caregivers had requested home visits because it was the most convenient location for them and the home environment is the most comfortable for the care recipient. In order to be consistent throughout the study, all subject dyads in both groups had baseline and final data collection done in the home setting. After informed consent but before random assignment, the project director (PD) or research assistant (RA) collected demographic and baseline data on the outcome variables and covariates. Then, the subject/dyad was randomized to either the intervention group or the control group. Upon learning the group assignment of the subject dyad, the investigator returned to his/her car and brought either the HST (booklet and sample items) or a standard patient information worksheet on home safety. We kept the two forms of patient education, HST and standard worksheet, separate so subject dyads would not see what the other group was receiving (single blinded). Subject dyads were told that at the end of their 3-month participation in the study they would be offered the alternative home safety education.

After randomization, the *intervention group* received the Home Safety Toolkit (HST), which has two components: (1) the booklet “Keep the Home Safe for a Person with Memory Loss” and (2) a canvas bag with low-cost sample items that were found in prior studies to be acceptable to families and effective to reduce risky behaviors and accidents. The HST booklet (Figure 1) was developed using well-established principles of reading comprehension and health literacy and was learner verified for attractiveness, comprehension, self-efficacy, and persuasiveness [30]. Caregivers in the intervention group had an opportunity to manipulate and practice using the home safety items. This was intended to increase self-efficacy for injury prevention and increase practical ability. Sample safety items included a motion sensor, slide bolt lock, stove knob covers, grab bar, rubber bathmat, medicine dispenser, smoke alarm, and general items such as a flashlight and nightlights (Table 1). 

The *control group* received the “Worksheet to Make the Home Safer,” a patient information sheet that has been used in clinical practice since 2003 [31]. The worksheet was used to standardize “customary care” among the 3 study sites, which was a requirement of the Institutional Review Board that was concerned about the subject dyads in the control group being at higher risk during the study period. The worksheet has accurate and practical recommendations for home safety in dementia of the Alzheimer's type (DAT) with a reading level of 5th to 6th grade; however, it is in a conventional format using words only and does not conform fully to the principles of health literacy. In addition, there are no sample safety items to stimulate self-efficacy.

Caregivers in both groups were given 15–20 minutes to look over the information and ask questions if they wished to clarify any of the provided information. Given that the intervention was designed to be self-directed and self-paced, there was no specific training provided by the research investigator to either the intervention or the control group, but the research investigator did answer questions initiated by the caregiver in both cohorts. During the study period of 3 months, the caregivers in both the intervention and control groups were called biweekly by the project director (PD) or research assistant (RA) to collect information on the Risky Behavior Questionnaire (RBQ). Interim (between the two home visits) data collection of risky behaviors and accidents was conducted to facilitate the caregivers' memory of “close calls” during the 3 months of the study. Close calls were behaviors by the person with dementia that worried the family caregiver and/or could have resulted in an injury, for example, lighting the stove or smoking. In our prior study, the use of a home safety log over the course of several months was difficult because risky behaviors were episodic and unpredictable. At 3 months after enrollment, a second home visit was conducted to collect Time 2 data. After this final data collection, caregivers were offered the alternative home safety education materials.

Fidelity to the protocol was achieved through training by the principal investigator ((PI) Kathy J. Horvath). The PI first demonstrated the home safety protocol and then the PD and RA each did a simulated home visit for a potential subject in each of the groups: intervention and control. Any observed differences between the PD and RA were discussed and resolved to achieve a standardized approach to the home safety protocol. At regularly scheduled team meetings, the protocol was discussed to review procedures and any new issues. A change in the RA incumbent midway through the study led to a demonstration and return demonstration by the PD and RA, respectively. 

### 2.3. Measures

*Demographics.* Baseline data included a conventional demographic sheet with care recipient's age, gender, and race and caregiver's age, gender, race, relationship to care recipient, and years of formal education. 

#### 2.3.1. Folstein Mini-Mental State Examination (MMSE) [26]

The MMSE is a commonly used and well-documented measure of global cognition. Although other brief cognitive assessments have shown better sensitivity and specificity for early cognitive impairment [32, 33], the MMSE was the most readily available instrument across the 3 study sites. Because the inclusion criteria required a confirmed diagnosis of Alzheimer's disease or a related disorder, the need to identify mild cognitive impairment was not required, and the MMSE was used as a measure of disease severity because of the availability of scores across study sites. For this study, the MMSE was used only to describe the sample and was not used for diagnostic purposes. Scores on the MMSE range from 0 to 30 with higher scores representing better cognition. Age and education level impact population-based norms for mean scores on the MMSE indicating that persons over the age of 60 with a minimum of 9–12 years of education have a mean score of 28 with a standard deviation of 1.7 [34]. Measuring MMSE in cohorts of older persons aged 75, 80, and 85 years or older indicates population norms as low as 22 in the oldest old with limited education [35]. 

#### 2.3.2. Physical Self-Maintenance Scale (PSMS) [36]

This scale measures 6 basic activities of daily living: toileting, feeding, dressing, grooming, physical ambulation, and bathing. The scale was developed and tested with a diverse sample of 265 subjects aged 60 or older, living in both institutional and community settings. The scale has good interrater reliability (*r* = 0.87; *r* = 0.91), and construct validity was demonstrated when compared to measures of mental status and behavioral adjustment (*r* = 0.38 and *r* = 0.38, resp.). In our prior studies, Cronbach's alpha for the scale was 0.81 and 0.85. Items are rated on a scale of 1 (can perform the task without any help) to 5 (person is totally dependent). Scores range from 6 to 30 with higher scores representing greater disability. Internal consistency reliability for this study was *a* = 0.855. 

#### 2.3.3. Functional Activities Questionnaire (FAQ) [37]

The FAQ measures instrumental activities of daily living (IADL) that are commonly performed by people living in the community. Initial testing of the FAQ demonstrated good sensitivity (0.85) and specificity (0.81) for detecting impairment which was comparable to the MMSE. Convergent validity was demonstrated through correlation with an independent neurologist's estimate of global functioning (*r* = −0.83), and concurrent validity was demonstrated with another measure of IADL (*r* = 0.72). Item to total correlations on the FAQ ranged from 0.80 to 0.90 [37]. There are 10 items, such as preparing a balanced meal and paying attention to TV, which are rated on a scale from normal = 0 to dependent = 3. The scores range from 0 to 30 with higher scores representing greater degrees of functional impairment. Internal consistency reliability for this study was *a* = 0.746. 

#### 2.3.4. Revised Scale for Caregiving Self-Efficacy [38]

This instrument is composed of 3 discrete subscales that are measured and interpreted separately, which is consistent with self-efficacy (SE) theory as being domain specific. This study used the subscale: SE for obtaining respite. This subscale is conceptually congruent with the Home Safety/Injury Model [21, 23]. One of the most unsafe environments is when the care receiver is left alone unsupervised. Obtaining respite for times when the caregiver has to do errands or other activities is an important task in order to avoid gaps in supervision. Additional items reflecting behaviors specific to home environmental safety were added in procedures common to self-efficacy measurement [39]. 

The additional 12 items asked caregivers to rate their confidence to prevent the high frequency/high severity risks for safety in the home of a person with DAT [15], for example, preventing the care receiver from using the stove or sharp knives and preventing the care receiver from leaving the house alone. Each of the total 17 items is rated as 0% confidence to 100% confidence. For statistical purposes, the summed score for all items was used in the analysis with potential scores ranging from 0 to 1700 with higher scores representing increased caregiver self-efficacy for home safety. Understanding the scores from a clinical perspective is discussed under Section 3. Internal consistency reliability for this study was *a* = 0.837 Time 1 and *a* = 0.803 Time 2. 

#### 2.3.5. MBRC Caregiver Strain Instrument [40]

The Margaret Blenkner Research Center Caregiver Strain Instrument is composed of four subscales which are scored separately. For this study, the subscale for health strain was used because the items capture potential negative consequences for the caregiver, such as physical health deterioration, more nervousness, and having less energy. Construct validity was demonstrated through factor analysis and convergent validity with other psychosocial measures. The scale consists of 5 items, which are rated as strongly agree (3), agree (2), disagree (1), and strongly disagree (0). Scores for the scale range from 0 to 15 with higher scores representing more strain. Internal consistency reliabilities for this study were *a* = 0.855 Time 1 and *a* = 0.842 Time 2. 

#### 2.3.6. Medical Outcomes Study Social Support Survey (MOS-SSS) [41]

This instrument was developed to measure functional support in contrast to structural support. Functional support is defined as the perception of the availability of interpersonal relationships for particular needs, such as preparing meals, helping with daily chores, and giving advice. The MOS-SSS consists of 19 items which are scored from 1 to 5 with 1 = “None of the Time” and 5 = “All of the Time.” The scale consists of 4 subscales which can be scored separately or as a total overall social support index. The overall index was used for this study. Internal consistency reliability for the overall index was Cronbach's *a* = 0.97. Construct validity was tested with product moment correlations with other measures of emotional and physical health [41]. The highest correlation was with a measure of loneliness, and the lowest correlation was with a measure of pain severity (*r* = −0.67; *r* = −0.19; *P* < 0.01). Scale items are averaged and then transformed so the possible range of scores is 0–100, with higher scores representing higher perceived social support. Internal consistency reliabilities for this study were *a* = 0.939 Time 1 and *a* = 0.948 Time 2. 

#### 2.3.7. Risky Behavior Questionnaire [15]

This tool was developed for use in the first home safety project in order to capture the behaviors that often lead to accidents and injuries. Content validity is derived from empirical evidence demonstrating the high severity and high frequency behaviors in persons with dementia of the Alzheimer's type (DAT) that could lead to an injury in the home. The number of risky behaviors and accidents is recorded at baseline and biweekly for 3 months. The additional biweekly interim data points were added to ensure good memory of “close calls,” the caregiver's perception of behavior that was risky but did not result in an accident or injury. For example, the care recipient tried to leave the house unsupervised but may not have exited. The baseline number of risky behaviors is summed for the month prior to the first home visit. The outcome measure for the variables of risky behaviors and accidents is the summed total of incidents that occurred during the 3-month duration of the study. Individual behaviors are not weighted because it is difficult to determine the severity of an incident if an actual injury does not occur. Based on our preliminary studies, we consider all of the risky behaviors as having an equal potential to cause harm, and thus the scores for risky behaviors and accidents are summed. Potential scores range from 0 to undetermined. The maximum score is undetermined because the measure represents the caregiver count of the number of times an incident occurred. In this study, postintervention sum scores ranged from 0 to 180 with a mean of 35.20 (SD 34.55). 

#### 2.3.8. Home Safety Checklist (HSC)

The HSC is used to measure the variable overall home safety. This tool was developed and used in preliminary studies with interrater reliabilities of 0.80–0.85, internal consistency reliability *a* = 0.84) and was sensitive to changes in home safety between enrollment (Time 1) and 6 months later (Time 2) with a statistically significant change in scores (*t* = 9.402; *P* ≤ 0.001). This relatively new tool was used because there is no other instrument available to measure home safety specifically. Lach et al. used a Home Safety Inventory as a daily log for caregivers to record risky behaviors, but the Home Safety Inventory is not a measurement tool [42]. Gitlin and colleagues developed and tested the Home Environmental Assessment Protocol (HEAP); however it consists of 192 items that assess not only hazards but also adaptations and comfort in the home [43]. Consequently, we believe that the HSC is the best tool to measure specifically the variable of home safety. The 64 items on the HSC reflect the specific recommendations in the educational materials that were given to both the intervention and the control groups. Each item is scored as NA = not applicable (e.g., for issues not included in all homes such as sliding glass doors), 1 = no safety issue; 2 = Safety modification implemented, 3 = traditional unsafe but not immediately threatening (e.g., hand rails on one side of stairs), and 4 = safety modification needed. Scores are summed and range from 5 to 256 with lower scores reflecting better home safety.

### 2.4. Data Analysis

The statistical package SPSS-PC was used to run all analyses [44]. Descriptive statistics were first computed on all study variables for all data collection points to examine the data for the presence of marked skewness, outliers, and systematic missing data. Hypotheses were tested using Multivariate Analysis of Covariance (MANCOVA) in order to test all outcome variables and covariates simultaneously. Prior to testing the hypotheses with MANCOVA, assumptions of normality of sampling distribution, homogeneity of variance-covariance matrices, linearity, multicollinearity, and singularity were checked. Tests for univariate and multivariate outliers were computed separately for each cell of the design in each hypothesis, and appropriate transformations or deletion of outlying cases was performed when needed using techniques specified in Tabachnick and Fidell [45]. 

## 3. Results

**Figure 2:**
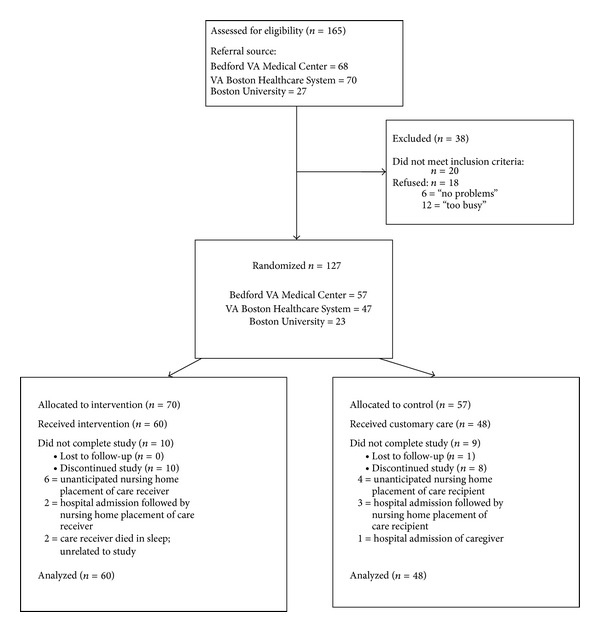
Flow diagram of referrals, randomization, and progress of the groups through the Home Safety Toolkit clinical trial.

We identified an initial population of 165 care recipient/caregiver dyads for the study, of which 127 dyads were enrolled. A CONSORT [46] flow diagram is depicted in Figure 2. Among the 38 dyads that did not enroll, 20 did not meet inclusion criteria, and 18 refused participation. 

Randomization of the sample used a blocked design, stratified by setting, in order to achieve balanced representation of each site in both the intervention and the control groups. Of the 127 dyads that enrolled, 70 were assigned to the intervention group and 57 to the control group using the sealed envelope method. A final sample of 108 dyads (60 in intervention group; 48 in control group) completed the study. No participant chose to withdraw, but rather, withdrawal was required because of changes in the status of either the care receiver or the caregiver that no longer met the inclusion criteria. Attrition was relatively low (15%) and dispersed evenly across intervention and control groups. We examined baseline characteristics (age, gender, years of caregiving, race, marital status, level of education, care receiver MMSE score, PSMS score, FAQ score, MBRC score, and MOS-SSS score) between the dropouts and completers using the appropriate statistics (chi-square for nominal data and *t*-tests with adjustments for type 1 error for continuous data) revealing no significant differences between the groups. The final sample had power of 0.82 or greater for all proposed analyses.

The sample is typical of care dyads for people with dementia of the Alzheimer's type (Table 2). The care recipients, people with DAT or a related disorder, are an older group with a wide range of disease severity. The caregivers are somewhat younger as a group, reflecting some primary caregivers who were adult children. Because two of the recruitment sites were veteran's administration facilities, the care recipients are more likely to be male with female caregivers; however there were no significant differences between the intervention and control groups on these demographic and disease severity measures.

Internal consistency reliabilities were acceptable for all instruments with Cronbach's alpha ranging from 0.746 to 0.948. Statistical analysis of the demographic variables and the dependent measures revealed that the variable of caregiver age correlated with the caregiver outcome measures in Hypothesis 1 (self-efficacy and caregiver strain), and it was therefore added to the analysis as a covariate. Years of education and social support did not correlate with the outcome variables in either hypothesis and were dropped from the analysis as covariates. Based on this preliminary data analysis, all MANCOVA analyses included the demographic variable of caregiver age and all Time 1 baseline measures set as covariates.

Table 3 reports the adjusted means for the outcome variables and the corrected MANCOVA model. Effect sizes were computed using Cohen's d and revealed effect sizes for home safety (0.21), caregiver self-efficacy (0.29), and caregiver strain (0.22). In relation to the significance of home safety as a problem area for persons with DAT living at home, these small effect sizes translate into clinically relevant findings. Making even modest improvements in home safety, caregiver self-efficacy and caregiver strain will make an impact on the often tentative circumstances of home caregiving for a person with DAT. Effect sizes must also be considered in the context of an educational intervention in the setting of a neurodegenerative chronic illness. The Institute of Educational Science suggests that effect sizes for educational interventions of 0.25 standard deviations or larger are considered substantively important [47]. Given that the analyses were adequately powered and statistical significance was found, the effect sizes in this study represent a reasonable magnitude for this intervention. 

The means for caregiver self-efficacy represent relatively high self-confidence on average. Converting the total sum to an average confidence in home safety, the Control group had 75% overall confidence in caregiving home safety, and the intervention group had 80% overall confidence in their ability to make a home safer (1305/1700 and 1350/1700, resp.). As discussed above, we believe that these small changes overall can have a significant clinical impact, but, more importantly, the use of the measurement instruments in a clinical setting is more relevant if the individual items are examined. An average score might represent high self-efficacy for preventing the person with DAT from using the stove or sharp knives but low self-efficacy for obtaining respite for the caregiver. Clinical interventions would be tailored to the caregiver's specific needs.

Caregiver strain is lower in the intervention group than in the control group, which represents less perceived strain in caregiving. The developers of the MBRC Caregiver Strain Instrument suggest a score of greater than 10 to indicate heightened risk requiring clinical investigation. Also a score can be used to assess change in the care situation over time. The group means in our study, which differ significantly in statistical analyses, indicate caregivers that are not on average at high risk. As with the other assessment instruments, however, in a clinical setting, an individual score that changes over time is important to monitor.

Overall home safety improved in the intervention group compared to the control group, with a lower score that represents fewer safety risks. With a range of potential scores on the Home Safety Checklist of 5–262, the means for the intervention group (133.58) and control group (129.32) reflect home environments that on average are at the midrange of environmental safety. Therefore, a small improvement (lower score) that is statistically significant still suggests that home safety modifications are an important part of caring for a person with DAT that requires ongoing monitoring.

The means for risky behaviors and accidents for the intervention (37.44) and control (33.95) groups indicate a low-moderate number of incidents on average (range = 0–180). This study was a small randomized trial to test an evidence-based intervention for efficacy. Although actual accidents and injuries can be devastating to a patient and family, in a sample of 108 dyads, the number is likely to be small in the 3 months of study participation. Therefore, risky behaviors, known to be a source of anxiety for the caregiver, were summed with accidents in order to have a measure for the outcome variable that could be analyzed statistically. Epidemiologic studies, reported in the literature review, establish the higher rate of accidents and injuries in this population of persons with DAT or a related disorder [12–14, 18, 19]. 

*Analysis Results for Hypothesis 1.* After controlling for the effects of caregiver age, and baseline measures of caregiver self-efficacy, baseline caregiver strain, and baseline home safety, caregivers who receive the Home Safety Toolkit will have improved home environmental safety higher postintervention self-efficacy, and lower postintervention caregiver strain than the control group which receives standard patient education. Hypothesis 1 was confirmed. Caregivers in the intervention group had significantly improved home environmental safety (*P* ≤ 0.000) higher caregiver self-efficacy (*P* ≤ 0.002), and lower caregiver strain (*P* ≤ 0.000) than caregivers in the control group. 

*Analysis Results for Hypothesis 2.* After controlling for the effects of caregiver age, baseline measures of caregiver self-efficacy, baseline caregiver strain, and baseline risky behaviors and accidents, care recipients who receive the Home Safety Toolkit will have fewer risky behaviors and accidents when compared to the care recipients in the control group who received standard patient education. Hypothesis 2 was confirmed. Care recipients in the intervention group had significantly less risky behaviors and accidents (*P* ≤ 0.000) than care recipients in the control group. 

## 4. Discussion

The Home Safety Toolkit is a practical intervention that significantly improved caregiver self-efficacy to prevent injury to the person with DAT living at home and reduced caregiver strain. The findings from this clinical trial support observations in clinical practice that, when caregivers are given an easy-to-read publication on making the home safer and an opportunity to practice home safety modifications, their competence and confidence as caregivers increase. 

The small effect sizes are a consideration regarding the value of implementing the Home Safety Toolkit intervention. We note, however, that our study actually tested two interventions: the Home Safety Toolkit with health literacy materials and sample items in contrast to a conventional patient information worksheet with written recommendations for making the home safer. Customary care in some dementia and geriatric clinics often does not include comprehensive home safety information, and the IRB at the participating sites were concerned about withholding safety information from a vulnerable group of patients and family caregivers. Thus, subject dyads in the control group received standardized home safety education that is unlikely in customary clinical care and decreased the size of the effects between the intervention and control groups. Nevertheless, the statistically significant results support the efficacy of the Home Safety Toolkit. 

 The findings have health policy implications with regard to funding for the Home Safety Toolkit sample items that promote caregiver self-efficacy. In order to standardize the intervention, all families in the intervention group were given the same type and amount of sample items with the exception of some large items such as a tub transfer bench. A large, special order item such as this was ordered based on individual need. Without the cost of the occasional tub bench, the cost per family for the Home Safety Toolkit booklet and sample items equals $210 USD. Some items were used immediately and some were kept for future use as the behavior of the person with dementia changed. 

A formal cost/benefit analysis was not undertaken because the study timeline would have been too long to capture health care utilization costs such as emergency room visits and hospitalization. Rather the focus of this current study was efficacy of the intervention. Nevertheless, most of the staff time in the study was related to research procedures such as informed consent and data collection and was included in the study budget. We specifically designed this intervention to be self-directed by the family caregiver, with an easy-to-read publication that would require little if any clinical staff time. A subsequent implementation research study, under development, is needed to analyze costs related to the Home Safety Toolkit. Further, the Home Safety Toolkit may be most appropriate in North America and Western European countries where housing structures are more similar to the housing arrangements of the study population. Two of the three study sites served the diverse urban population of Boston, Massachusetts, but we did not obtain income data from the participants and did not design the study to test for the potential effects of socioeconomic status on the study outcomes. However, we were surprised that years of education and perceived social support did not correlate with the outcome variables and therefore were not entered into the adjusted MANCOVA model. When these variables, often associated with socioeconomic status, were successfully randomized between the two groups, the Home Safety Toolkit still was effective to increase caregiver self-efficacy and home safety and decrease caregiver strain and care recipient risky behaviors and accidents. 

*Study Limitations.* Two of the participating facilities are US Department of Veterans Affairs medical centers, and therefore the study sample has few female care recipients and few male caregivers. Therefore, the potential for gender differences in the outcome variables was not tested. In a recent publication, male caregivers of a person with dementia reported less caregiver burden than female caregivers, even in situations where the care recipient was more impaired [48]. Future studies of the potential effects of gender on interventions such as the Home Safety Toolkit should be undertaken. 

The study design was single blinded in that the subjects did not know which group they had been assigned to randomly, but the project director and research assistants were aware of group assignment. Double blinding, as is done in drug clinical trials, could not be done, because the Home Safety Toolkit could not be disguised; the sample home safety items were an obvious sign of group assignment. Thus, there may have been bias in the data collection following randomization. Baseline data were collected before group assignment, as one control on potential bias, and procedures were reviewed frequently between the PI and statistician (who were blinded to both groups) and the project staff who were collecting data.

## 5. Conclusion

The Home Safety Toolkit (HST) utilized principles of health literacy and self-efficacy to activate the primary family caregiver to manage the high frequency and high severity home safety issues for a person with dementia of the Alzheimer's type (DAT). The intervention is consistent with new models of patient-centered care where the patient and family caregiver are full partners with professional providers. In DAT, in particular, with the amount of unpaid care provided by family caregivers, this partnership is indispensable to the well-being of the person with DAT. This relatively low-technology evidence-based intervention now requires the implementation of strategies to enable primary care providers to prescribe a Home Safety Toolkit for persons with DAT and their family caregivers. Addressing policy issues regarding how the sample safety items in the Home Safety Toolkit will be stocked and incorporated into operational budgets will also contribute to successful implementation and sustainability. 

## Figures and Tables

**Table 1:** Home safety items.

Items	Cost (USD)
Motion sensor with battery	28.49
Canvas bag	2.50
Smoke alarm	15.29
Colored duct tape (2 inch)	4.00
Night lights (with photo sensor)	4.95
Stove knob covers	6.29
Grab bar (18 inch)	23.75
Slide bolt lock	10.49
Medicine case	6.26
Keyed doorknob	15.99
Surge protector	7.99
Carbon monoxide alarm	25.99
Flashlight with batteries	5.98
Hand-held shower	24.00
Rubber bath mat (machine washable)	14.39
Cabinet slide lock	2.00
Home safety workbook	10.00

Total	208.35

**Table 2:** Sample characteristics.

	Control (*n* = 48)	Experimental (*n* = 60)
Caregiver age	69.4 (12.9)	70.6 (11.4)
Care recipient age	80.9 (7.2)	80.4 (6.7)
Mini-Mental State Examination	13.0 (6.9)	12.4 (6.6)
Physical self-maintenance scale	15.1 (4.0)	14.6 (4.3)
Functional activities questionnaire	26.4 (3.1)	25.8 (4.1)
Gender of caregiver	79.2% female	81.7% female
Gender of care receiver	87.5% male	86.7% male
Married	68.8%	71.7%
Caucasian	92.7%	88.3%

**Table 3:** Corrected model MANCOVA: tests of between-subjects effects*.

	Group means (SD)		Type III sum of squares	df.	Mean square	*F*	Sig.	Partial Eta. squared	Noncent. parameter	Observed power
	Control (*n* = 48)	Intervention (*n* = 60)								
Caregiver self-efficacy	1305.65 (203. 36)	1350.30 (197.18)	2633427.731	45	58520.616	2.189	0.002	0.614	98.508	0.999
Caregiver strain	6.96 (3.86)	5.95 (3.17)	904.965	45	20.110	2.976	0.000	0.684	133.936	1.000
Home safety	133.58 (20.15)	129.32 (20.02)	28004.977	45	622.333	2.537	0.000	0.648	114.177	1.000
Risky behaviors and accidents	37.44 (37.04)	33.95 (32.57)	97564.778	45	2168.106	4.504	0.000	0.7666	202.686	1.000

*Covariates include baseline measures of outcome variables and age of caregiver.
